# Molluscicidal and Schistosomicidal Activities of 2-(1*H*-Pyrazol-1-*yl*)-1,3,4-thiadiazole Derivatives

**DOI:** 10.3390/ph18030429

**Published:** 2025-03-18

**Authors:** Leonardo da Silva Rangel, Daniel Tadeu Gomes Gonzaga, Ana Cláudia Rodrigues da Silva, Natalia Lindmar von Ranke, Carlos Rangel Rodrigues, José Augusto Albuquerque dos Santos, Nubia Boechat, Keyla Nunes Farias Gomes, Guilherme Pegas Teixeira, Robson Xavier Faria

**Affiliations:** 1 Laboratory for Evaluation and Promotion of Evaluation and Promotion of Environmental Health (LAPSA), Oswaldo Cruz Institute, Oswaldo Cruz Foundation, Rio de Janeiro 21040-900, Brazil; leonardo.rangel.farmacia@gmail.com (L.d.S.R.); kfarias1618@gmail.com (K.N.F.G.); gpegas67@gmail.com (G.P.T.); 2 Department of Pharmacy, West Zone Campus, State University of Rio de Janeiro (UERJ), Rio de Janeiro 20550-013, Brazil; daniel.gonzaga@uerj.br; 3 Laboratory of Molecular Modeling and QSAR (Mod Mol QSAR), Faculty of Pharmacy, Federal University of Rio de Janeiro, Rio de Janeiro 21941-599, Brazil; nataliavonranke@outlook.com (N.L.v.R.);; 4 Laboratory of Drug Synthesis-LASFAR-Farmanguinhos, Fiocruz 21041-250, Brazil; 5 Postgraduate Program in Plant Biotechnology and Bioprocesses, Center of Health Sciences, Federal University of Rio de Janeiro, University City, Carlos Chagas Filho Avenue 373, Rio de Janeiro 21941-902, Brazil

**Keywords:** *Biomphalaria glabrata*, schistosomiasis, 1,3,4-thiadiazole, synthetic molecules

## Abstract

**Background/objectives:** Schistosomiasis is caused by flatworms of the genus *Schistosoma*, for which mollusks of the genus *Biomphalaria* are intermediate hosts. Niclosamide (NCL) is a molluscicide recommended by the World Health Organization (WHO) for control of *Biomphalaria*. Although effective, it is expensive and environmentally toxic, which raises concerns regarding its widespread use. As a result, we explored new synthetic substances as alternative strategies for controlling *Biomphalaria glabrata*. We evaluated the molluscicidal activity of 2-(1***H***-py-razol-1-***yl***)-1,3,4-thiadiazole and 2-(4,5-dihydro-1***H***-pyrazol-1-***yl***)-1,3,4-thiadiazole derivatives against *B. glabrata* snails and embryos, as well as *Schistosoma* cercariae (infective larvae). **Methods:** Adult and young snails were added to 24-well plates containing 20 synthetic compounds from the PDAN series for initial screening over 96 h at a concentration of 100 ppm. Water and NCL (2 ppm) were used as the negative and positive controls, respectively. Active compounds in the adult *B. glabrata* assay were selected for the tests vs. embryos and cercariae. **Results:** In the initial screen, only PDAN 52 (63 ± 4%) and 79 (12 ± 3%) showed molluscicidal activity at a concentration of 100 ppm up to 48 h. Consequently, we selected only PDAN 52. The LC_50_ value found in the tests on embryos after 24 h of treatment was 20 ± 2 ppm and, after 48 h, it was 4 ± 0.5 ppm. Against cercariae, we measured an LC_50_ value of 68 ± 5 ppm after 4 h of treatment. PDAN 52 did not induce marked toxicity against a second mollusk, *Physella acuta*, after 48 h of exposure. **Conclusions:** We highlight the promising molluscicidal activity of PDAN 52 against different developmental stages of the mollusk, *B. glabrata*, as well the infective larvae of *Schistosoma mansoni*.

## 1. Introduction

Schistosomiasis is a parasitic disease for which one of the etiological agents is the helminth *Schistosoma mansoni* (*S. mansoni*). *S. mansoni* has a complex life cycle, presenting different developmental stages depending on the infected host (intermediate or definitive) [1]. According to the World Health Organization (WHO), schistosomiasis occurs in approximately 78 countries and is one of the most neglected diseases in the world. In 2021, at least 251.4 million people required preventive treatment [2]. In addition, approximately 600 million individuals live in risk areas, which makes prevention and control of the disease a global priority [3].

**Figure 1:**
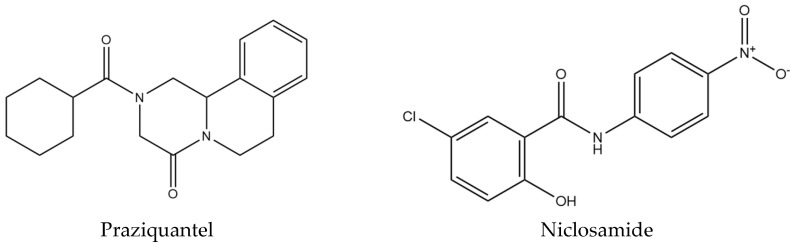
Standard drugs for schistosomiasis treatment (praziquantel) and molluscicidal control (niclosamide).

Schistosomiasis infection represents a serious public health problem, since, to date, there is no effective vaccine against this pathogen. The disease is treated with the drug praziquantel (Figure 1), which has proven to be effective and safe in combating the parasitosis. However, although it is effective in treatment, praziquantel is not a definitive solution for preventing the disease. Therefore, one of the strategies adopted to control schistosomiasis is vector control, aiming to reduce the transmission of the parasite by controlling intermediate hosts [4,5].

For the disease transmission cycle to occur, the parasite uses snails of the genus *Biomphalaria* as an intermediate host, with *Biomphalaria glabrata* (*B. glabrata*) (Gastropoda: Planorbidae) being the main species associated with the transmission of schistosomiasis in Brazil, due to the high levels of infection and its wide distribution in the country [6]. Niclosamide (NCL), recommended by the WHO, is widely used as a pesticide for this purpose (Figure 1) [7]. Although NCL is effective in controlling snails, its application can result in toxicity to nontarget species (*Danio rerio*), generating environmental impacts [3,8,9]. Therefore, research and development of new molluscicidal substances that are safe, low-cost, and selective are essential to improve the control of schistosomiasis and minimize environmental damage.

The 2-(1***H***-pyrazol-1-***yl***)-1,3,4-thiadiazole derivatives consist of linking the pyrazole nucleus with the 1,3,4-thiadiazole group, and 2-(4,5-dihydro-1***H***-pyrazol-1-***yl***)-1,3,4-thiadiazole derivatives are the linking of the 4,5-dihydro-pyrazole nucleus with the 1,3,4-thiadiazole group. They were obtained exclusively by synthetic means in a methodology presented in a previously published work [10]. We performed tests with 20 prototypes of the PDAN series and evaluated its biological effect against *B. glabrata*, embryo different forms, and the cercariae of *S. mansoni*. Additionally, we evaluated the toxic effect of the promisor prototype against nontarget snails and the prediction of environmental and human toxicity using in silico analysis.

## 2. Results

### 2.1. Moluscicidal Activity

We investigated the molluscicide PDAN series (100 ppm) activity on adult *B. glabrata* for 96 h. Among all molecules, only PDAN 52 and 79 caused mortality at 96 h. PDAN 52 treatments gave a mortality of 15 ± 3% after 24 h and 63 ± 4% after 48 h. However, PDAN 79 promoted a slight effect at 24 h (7 ± 1%) and 48 h (12 ± 3%). Therefore, we selected PDAN 52 for subsequent analysis. We observed a linear relationship between the concentration of PDAN 52 and the mortality rate of adult *B. glabrata* mollusks (Table 1).

**Figure 2:**
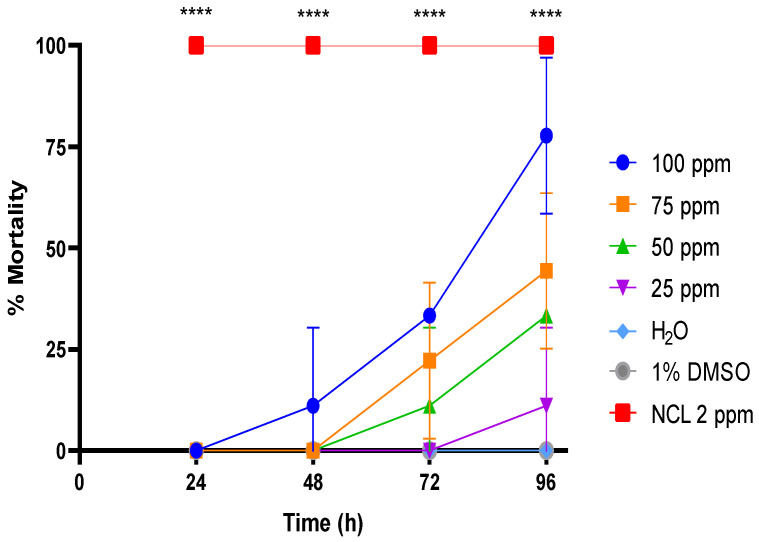
Mortality rate of PDAN52 on the adult snail *Biomphalaria glabrata* exposed for 96 h. The lethal concentration 50 (LC_50_) was 79.3 ppm and the lethal concentration 90 (LC_90_) was 99.2 ppm at 96 h. This experiment was performed in triplicate on at least 3 different days (*n*= 9). The results expressed in the graph represent the mean ± standard error. **** *p* < 0.0001.

Exposure of adult *B. glabrata* mollusks to concentrations of PDAN 52 presented LC_50_ and LC_90_ values determined 96 h after exposure of 79.3 ± 7 ppm and 99.22 ± 6.5 ppm, respectively (Table 2). The treatment with concentrations ranging from 25 to 100 ppm did not reach 100% mortality in 24 or 48 h (Figure 2). The maximal effect of PDAN 52 only occurred after 96 h. However, PDAN 79 maintained the toxic effect, in turn, of 15%. This result confirms the molluscicidal potential of PDAN 52 against adult mollusks of *B. glabrata*.

**Figure 3:**
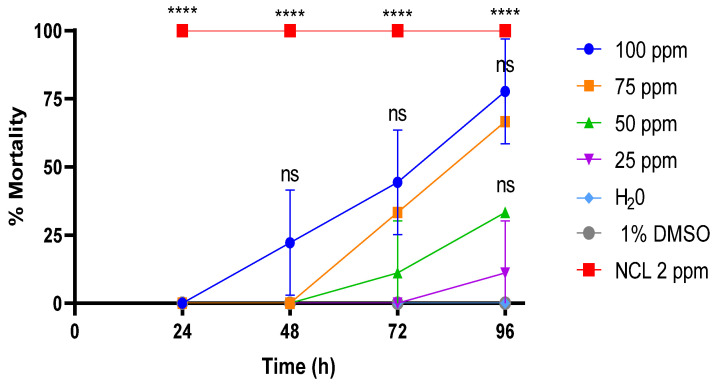
Mortality rate of PDAN52 on the young snail *Biomphalaria glabrata* exposed for 96 h. The lethal concentration 50 (LC_50_) was 66.7 ppm and the lethal concentration 90 (LC_90_) was 114.4 ppm at 96 h. This experiment was performed in triplicate on at least 3 different days (*n*= 9). The results expressed in the graph represent the mean ± standard error. **** *p* < 0.0001. ns = not significant.

Experiments in young *B. glabrata* snails with PDAN 52 did not reproduce the effect observed in adults (Figure 3). Only the concentration of 100 ppm gave a mortality of 17 ± 1% after 48 h, and continuous exposure until 96 h increased the mortality by 28 ± 2.6%. The exposure of young *B. glabrata* mollusks to PDAN 52 concentrations resulted in LC_50_ and LC_90_ values of 66.7 ± 3.5 ppm and 114.4 ± 3.5 ppm, respectively, after 96 h of exposure (Table 3).

**Figure 4:**
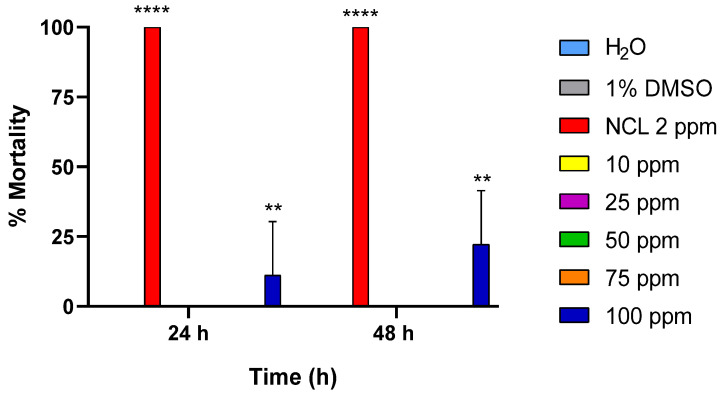
Mortality rate of *Physella acuta* mollusks affected by PDAN 52 exposed for 48 h. This experiment was performed in triplicate on at least 3 different days (*n* = 9). The results expressed in the graph represent the mean ± standard error. ** *p* = 0.017; **** *p* < 0.0001.

Additionally, we performed experiments on PDAN 52 using the mollusk *P. acuta*. PDAN 52 did not cause significant alterations to *P. acuta* after treatment with concentrations ranging from 10 to 75 ppm. However, the concentration of 100 ppm presented a slight difference in comparison to the negative control groups, causing 11.11% and 22.22% mortality in *P. acuta* after 24 and 48 h, respectively. Therefore, these results reinforce the high selectivity of PDAN 52 against *B. glabrata* (Figure 4).

**Figure 5:**
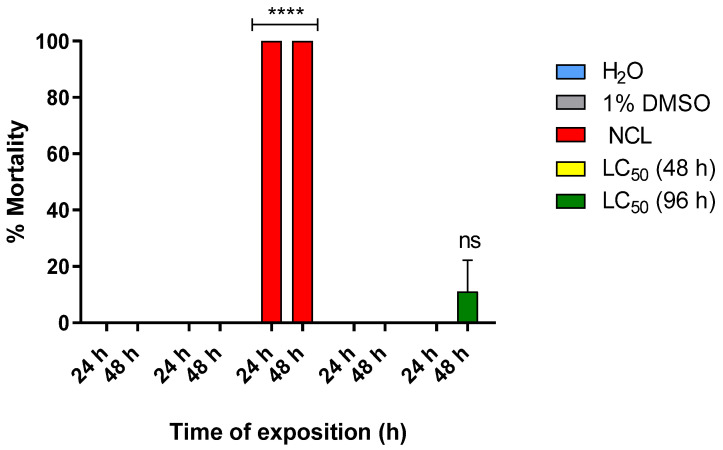
Mortality rate effect of sublethal concentration 50 of 48 h and 96 h of PDAN 52 on *B. tenagophila*. This experiment was performed in triplicate on at least 3 different days (*n* = 9). The results expressed in the graph represent the mean ± standard error. **** *p* < 0.0001; ns = not significant.

The acute toxicity test was carried out on the species *B. tenagophila*, using the sublethal concentration 50 for 48 h and 96 h obtained in the experiment with *B. glabrata*. It was possible to observe a low lethality of 11.11% at sublethal concentration 50 for 96 h (Figure 5).

### 2.2. Embryotoxicity

**Figure 6:**
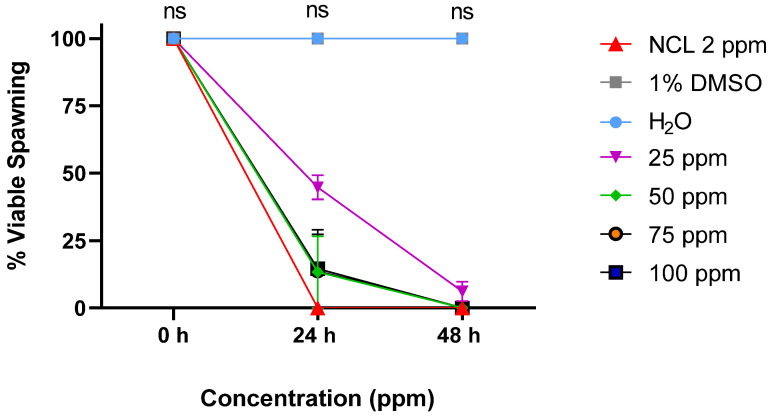
Effect of PDAN 52 on *B. glabrata* embryos in the period from 24 to 48 h. This experiment was performed in triplicate on at least 3 different days (*n* = 60). The results expressed in the graph represent the mean ± standard error. ns = not significant.

PDAN 52 caused significant alterations in *B. glabrata* embryos at different concentrations, resulting in 100% mortality after 48 h from the 50 ppm concentration (Figure 6). These results highlight the high toxicity and potent activity of PDAN 52 against *B. glabrata* embryos.

### 2.3. Cercaricidal Activity

**Figure 7:**
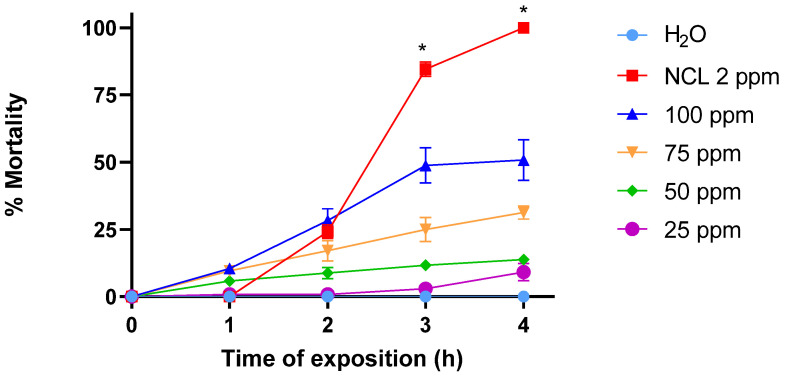
PDAN 52 activity against *S. mansoni* cercariae for 4 h. The test was performed in triplicate on different days using a range of 80 cercariae per well during the testing of the samples (*n* = 240). These data are expressed as the mean ± standard error. * *p* = 0.0273.

The crescent concentrations of PDAN 52 caused 50% lethality to *S. mansoni* cercariae after 4 h of exposure (Figure 7) when compared to the negative control groups. The LC_10_, LC_50_ and LC_90_ for PDAN 52 were 24.2 ± 2.8 ppm, 68 ± 5 ppm and 133.4 ± 7.3 ppm, respectively, after 4 h (Table 4). These data indicated that PDAN 52 is a moderate cercaricide agent.

### 2.4. Toxicity

**Figure 8:**
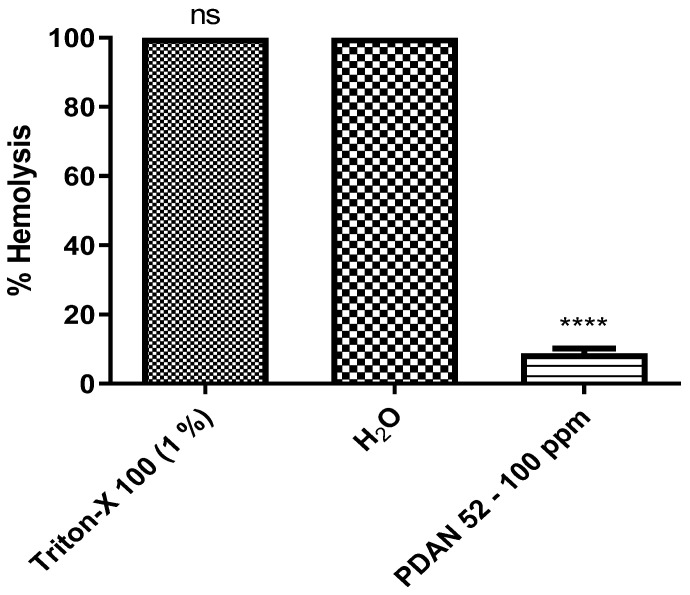
PDAN52 activity against red blood cell viability after 4 h using a concentration of 100 ppm (*n* = 10). These data are expressed as mean ± standard error. **** *p* < 0.0001. ns = not significant.

The possible toxicity caused by PDAN 52 in mammals was investigated using an ex vivo model with red cells and in vivo oral administration in mice. Treatment with 100 ppm of PDAN 52 for 4 h did not cause hemolysis above 10% (Figure 8). Most new drug candidates exhibit hemolytic activity until 10% is tolerable [11]. These data suggest that PDAN 52 is considered secure.

Additionally, we orally administered 1000 mg/kg PDAN 52 to mice and evaluated the mortality and behavioral changes. As a result, we did not observe toxicity for this substance after 24 h of administration.

### 2.5. In Silico Assay

The ecotoxicological assessment revealed distinct profiles for the PDAN 52 compound compared to NCL. While both compounds were predicted to exhibit low biodegradability, PDAN 52 demonstrated a higher bioconcentration potential than NCL.

In terms of aquatic toxicity, PDAN 52 was predicted to be less toxic than NCL toward *Tetrahymena pyriformis* (*T. pyriformis*). However, when considering *Daphnia magna* (*D. magna*) and minnows, PDAN 52 exhibited higher toxicity than NCL, particularly toward *D. magna*. This discrepancy suggests that PDAN 52 may have a different impact on aquatic toxicity compared to the well-known NCL. Regarding the potential interaction with endocrine receptors, PDAN 52 did not demonstrate any activity on the tested androgen and estrogen receptors. In contrast, NCL exhibited toxicity toward androgen receptors. These findings highlight a notable difference between PDAN 52 and NCL concerning their effects on endocrine receptors.

Finally, concerning the overall toxicological risk for mammals, both compounds presented a similar risk profile. Table 5 provides an overview of the overall ecotoxicological risk assessment.

## 3. Discussion

To control schistosomiasis, mass chemotherapy using praziquantel remains the most effective method to treat this disease. However, control of vector mollusks has received worldwide attention in recent years by national and international control programs [12,13]. The PDAN series was tested in this study to investigate its molluscicidal, embryocidal, and cercaricidal activities and environmental toxicity using *P. acuta*.

Previously, some authors evaluated the molluscicidal activity of thiophene, thiadiazole, and pyrazole derivatives against mollusks of the *Biomphalaria* genus. Thus, Fadda and collaborators tested eight compounds against *Biomphalaria alexandrina* (*B. alexandrina*) and described toxic activity after 24 h with LC_90_ values ranging from 9 to 23 ppm [14]. They reported thiadiazole derivatives with higher activity than thiophene derivatives, probably due to the strong electron-donating effect of the thiadiazole ring. Another group studied the molluscicidal activity of thiadiazoles and thiadiazines against *B. alexandrina* [15,16]. Only two substances caused 100% mortality at 10 ppm after 24 h.

A series of pyrazoles was examined concerning the viability of *B. alexandrina* snails after 24 h of exposure. Four substances exhibited satisfactory molluscicidal activity with LC_90_ ≥ 14 ppm [17,18]. Curiously, a study on the metabolic content of the tunicate *Polycarpaaurata* isolated two novel alkaloids, polyaurines A and B. They determined the presence of a 1,2,4-thiadiazole ring in polyaurine B. Both substances were not toxic to mammalian cells. However, polyaurine B has not affected *S. Mansoni* [19].

PDAN 52 massively affects the survival of *B. glabrata* embryos, eliminating all embryos within 48 h, with a lethal concentration of ~17 ppm. The concentration of 100 ppm of PDAN 52 caused 50% lethality to *S. mansoni* cercariae after 4 h of exposure when compared to the negative control group. These data are extremely relevant as a function of the absence of other data exploring thiadiazole derivatives acting against *S. mansoni* cercarie.

Li and collaborators realized a high-throughput screening (HTS) assay in vitro against thioredoxin glutathione reductase (TGR) from *S. mansoni* (SmTGR). The authors tested 59,360 synthetic compounds. Approximately 928 or 1.56% of the substances inhibited SmTGR activity by more than 90% after treatment with 10 μM in the primary screening. Among these, 74 of them (17 or 0.12%) were confirmed and showed a concentration-dependent inhibitory result against SmTGR, including a 2,5-dithio-1,3,4-thiadiazole (e.g., WNN0192-H003) [20].

A study evaluated novel compounds that selectively inhibit triosephosphate isomerase (TIM), an enzyme of the glycolytic pathway, focusing on *Fasciola hepatica* TIM (FhTIM). Sulfonyl-1,2,4-thiadiazole (compound 187) inhibited FhTIM, exhibited low toxicity in vitro and lacked acute toxicity in mice and various developmental stages of *S. mansoni* [21].

The results of molluscicide trials with PDAN 52 revealed that, although the substance caused low lethality in both adult and juvenile mollusks after 48 h of exposure, the mortality rate increased significantly after 96 h, reaching approximately 63%. These data suggest that, over time, PDAN 52 presents increasing efficacy, possibly due to the accumulation of toxic effects throughout the exposure period.

NCL, in turn, is the only synthetic molluscicide approved by the WHO and is still widely used to control mollusks that are hosts of Schistosoma spp. Despite its proven efficacy, niclosamide has been associated with environmental concerns, given its toxicity to nontarget organisms, in addition to cases of resistance emerging among mollusk populations. These challenges have led to the urgent need to seek new compounds that can replace or complement the action of niclosamide, without causing the same environmental impacts or favoring the development of resistance in the target mollusk [3,22]. In this context, PDAN 52 presents itself as a possible alternative, with its selectivity profile and increasing efficacy over time.

When comparing niclosamide with PDAN 52, it is essential to consider both the economic and scientific aspects. Niclosamide is a substance that has been extensively optimized for large-scale production, which results in significantly lower costs. On the other hand, PDAN 52, which is still in the experimental phase, has higher costs due to the absence of an optimized production process. Thus, the disparity in costs between the two substances is directly related to the stage of development of each. PDAN 52, in order to become a viable alternative in terms of cost–benefit, still requires improvements in the production process [23,24].

In terms of prices, in 2016, the international value of niclosamide varied between USD 14 and 30 per kilo, depending on the destination and the quantity purchased [25]. In the current market, at Merck, the price of niclosamide is USD 248.07 for 50 g and USD 951.75 for 250 g. If we consider an estimate for PDAN 52, without optimizing the production process, the cost would be approximately USD 46.14 per 5 g [26]. This reinforces the need for optimization in the PDAN 52 production process to reduce its costs.

The ecotoxicological assay is a suitable tool that provides detailed information for the execution of aquatic protection policies [27]. It is essential to evaluate the environmental effects of commercial chemicals before releasing them into the market, in part to prepare for accidental release from production plants, with exposure to aquatic species being of regulatory concern. Therefore, standard experimental protocols have been established by the chemical industry, pharmaceutical companies and government agencies to test chemicals for their toxic potential [28,29].

Although experimental protocols for toxicity testing have been developed for many years, computational chemical toxicology continues to be a viable approach to reduce both the amount of effort and the cost of experimental toxicity assessment. In this regard, we used the ADMET Predictor (Simulation Plus) to predict the environmental effects of the tested compounds.

Regarding the bioconcentration factor (BCF), PDAN 52 presented a value slightly higher than that of NCL. The EU REACH considers a substance with a BCF greater than 2000 to be regarded as bioaccumulative and a substance with a BCF greater than 5000 to be regarded as very bioaccumulative. In the United States, a substance is considered not bioaccumulative if it has a BCF less than 1000, bioaccumulative if it has a BCF from 1000 to 5000, and very bioaccumulative if it has a BCF greater than 5000. Therefore, PDAN 52 is not predicted to be a bioaccumulative compound. PDAN 52 and NCL are predicted not to be biodegradable. Therefore, attention must be paid to the amount of waste generated in the environment and its potential harm [30].

Aquatic toxicity was predicted for three species at different trophic levels: *T. pyriformis*, *D. magna*, and *P. promelas*. PDAN 52 was predicted to have *T. pyriformis* toxicity sensitivities similar to those of NCL. However, PDAN 52 is predicted to be 30 times more toxic to *D. magna* than NCL [22,30].

The endocrine toxicological results indicated that the PDAN 52 compound did not present the potential to interact with the tested receptors. This suggests a low endocrine toxicological event of this compound when compared to NCL. Indeed, some recent works have reported the inhibition of androgen receptors by NCL [31,32,33]. Finally, PDAN 52 presented a lower overall toxicology risk, similar to NCL.

Additionally, this series of thiadiazoles was tested as an inhibitor of a membrane receptor related to inflammation [10]. PDAN 52, prototype 9c, was not toxic to mouse peritoneal macrophages. We confirmed this lack of toxic effect in ex vivo red cells and in vivo in mice.

## 4. Material and Methods

### 4.1. Chemistry

The synthetic compounds were obtained in collaboration with Prof. Gonzaga (Department of Pharmacy, UERJ, Rio de Janeiro, Brazil) and were prepared as previously reported [10].

### 4.2. Bioassays

#### 4.2.1. Molluscicidal Assays

Initially, we screened the molluscicidal biological activity of 20 candidate substances, using a standard initial concentration of 100 ppm, according to the methodology of Santos et al. (2017) [34]. For each substance, 9 *B. glabrata* mollusks (10–12 mm) were used, with 3 animals per replicate, and the exposure lasted 96 h. The evaluation was based on the absence of shell retraction and the release of hemolymph [35]. After the initial screening, the most active candidates were selected, and the number of concentrations tested was expanded to 25 ppm, 50 ppm, 75 ppm and 100 ppm, maintaining the number of 9 mollusks (3 per replicate), as described in the methodology of Santos et al. [34].

Adult and juvenile *B. glabrata* mollusks (shell diameter between 6 and 8 mm for adults and between 10 and 12 mm for juveniles) were obtained from the Environmental Health Assessment and Promotion Laboratory of the Oswaldo Cruz Institute. The specimens were allocated into groups of three and individually exposed to 24-well plates [34] containing 2 mL of aqueous solution with PDANs at concentrations of 25 ppm, 50 ppm, 75 ppm and 100 ppm for 24 and 48 h. The compound NCL at 2 ppm was used as a positive control, while the negative control wells contained only distilled water. Mortality was assessed from 24 h to 96 h, with the evaluation criteria being the absence of shell retraction and the release of hemolymph [35].

To assess environmental toxicity, we used *Physella acuta* (*P. acuta*) mollusks, which, despite being invasive, are sensitive to exposure to chemical substances. Tests were also performed with mollusks of the species *Biomphalaria tenagophila* (*B. tenagophila*), using the same parameters described above.

#### 4.2.2. Evaluation of Ovicidal Activity

The ovicidal activity assay was performed using 3 × 3 cm Styrofoam plates, which were placed at the bottom of the rearing tank to facilitate egg deposition. At the beginning of the experiment, the plates were introduced into the tanks containing *B. glabrata* to allow oviposition. After 48 h, the egg capsules adhered to the plates were carefully removed and transferred to 24-well plates, following the methodology adapted from Araújo et al. [36]. Egg counts were performed using a stereomicroscope, with 10× magnification. Initially, the number of viable eggs was counted (time zero), and then 1000 μL of the PDAN 52 solution was added to the wells at concentrations of 25 ppm, 50 ppm, 75 ppm and 100 ppm. After 24 and 48 h of exposure, viable egg counts were repeated to evaluate the ovicidal activity of the compound.

#### 4.2.3. Evaluation of Cercarial Activity

*Schistosoma mansoni* cercariae were obtained from infected *B. glabrata* snails, kept in 10 mL glass beakers with dechlorinated water and exposed to 60 W incandescent lamps positioned at a height of 30 cm. Exposure to light and heat induced the spontaneous release of cercariae, due to the phototropism and thermotropism characteristic of this larval stage. For initial quantification, the concentration of *S. mansoni* cercariae in the suspension was estimated using 20 μL of Lugol’s iodine and counted under a stereomicroscope. The suspension containing cercariae was distributed in 24-well plates [34], with 1000 μL of cercariae suspension being added to each well. Then, 1000 μL of PDAN 52, at concentrations of 25 ppm, 50 ppm, 75 ppm and 100 ppm, was added to each well. To assess the viability of the cercariae, 20 μL of 0.1% Trypan Blue dye was added, which allows the differentiation between viable cercariae (without color) and dead cercariae (bluish color). Mortality was assessed after 1, 2, 3 and 4 h of exposure, counting the dead cercariae under a stereomicroscope.

#### 4.2.4. Toxicity Hemocompatibility

The toxicity of PDAN 52 was evaluated by the hemocompatibility test, according to Bauer and collaborators’ method, with modifications [12]. The compound (100 ppm) or saline (negative control) was incubated with a 13% (*v*/*v*) red blood cell suspension for 3 h at 37 °C. Then, the samples were centrifuged for 3 min at 1800 rpm, and the lysis of the cells was detected by measuring hemoglobin at an absorbance of 578 nm using a microplate reader (SpectraMax, Model M4, Molecular Devices, San Jose, CA, USA). One hundred percent hemolysis (positive control) was achieved by adding Triton X-100 (1%, *v*/*v*) or water to the red blood cell suspension.

#### 4.2.5. Single-Dose Toxicity

PDAN 52 toxicity was evaluated by the in vivo test, according to ANVISA et al. [37], with modifications. PDAN 52 (1000 mg/kg), saline solution or DMSO was injected intraperitoneally (i.p.) into the abdominal region of the mice. Then, behavior and mortality were observed for 24 h. Mice experiments were carried out under license L039-2016.

### 4.3. Statistical Analysis

Statistical analysis of the experiments was performed using the Prism 8 GraphPad program (GraphPad software 8.0) using one-way ANOVA with a significance level of *p* < 0.0001 compared with the negative control. Lethal concentrations (LC_10_, LC_50_ and LC_90_) were calculated using the Statgraphics Program 19.5.01.

### 4.4. In Silico Assay

We performed the prediction of the ecotoxicity profile using ADMET Predictor™ (version 9.5, Simulations Plus, Lancaster, CA, USA). The PDAN 52 structure was compiled in the format of the simplified molecular-input line-entry system (SMILES) and entered ADMET Predictor™. The toxicological endpoints investigated included bioconcentration, biodegradation, aquatic toxicity (Tetrahymena pyriformis, water flea (Daphnia), and Fathead minnow), and endocrine toxicity (estrogen/androgen receptor). Furthermore, we evaluated the toxicological risk associated with the compound based on six key characteristics: potential hERG liability, acute toxicity in rats, carcinogenicity in chronic rat studies, carcinogenicity in chronic mouse studies, hepatotoxicity, and mutagenicity.

## 5. Conclusions

The results obtained from the exposure of the test organisms allow us to conclude that PDAN 52 showed moderate molluscicidal activity against *B. glabrata* mollusks, both adults and juveniles, being able to eliminate approximately 63% of the population. At the same time, it was not toxic to nontarget organisms present in the aquatic environment, as evidenced in the test with *P. acuta*. Furthermore, PDAN 52 demonstrated high efficacy against *B. glabrata* embryos, eliminating 100% of the exposed embryos after 48 h. Regarding cercariae, PDAN 52 showed moderate lethality, eliminating 50% of the parasite population in just 4 h of exposure. It is important to highlight that the compound showed no toxicity to mammalian tissues. Thus, PDAN 52 presents itself as a promising alternative for the control of schistosomiasis, in addition to serving as a basis for the development of new analogues with molluscicidal activity.

## Figures and Tables

**Table 1:** Chemical structure of synthetic derivatives and molluscicide activity results.

Compounds	Structure	% Molluscicide Activity in 96 h
**PDAN52**	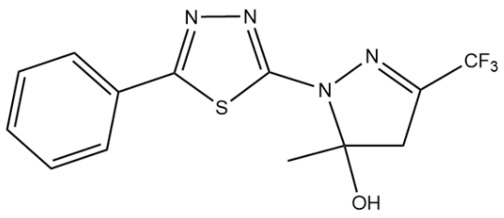	63 ± 4
**PDAN79**	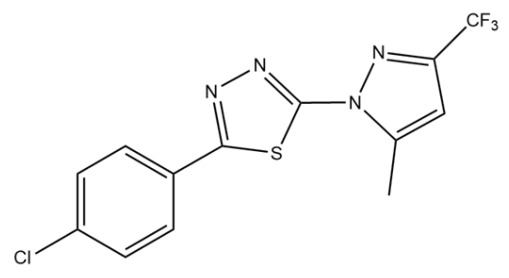	12 ± 3

**Table 2:** Molluscicidal effect of PDAN 52 against adult *B. glabrata*.

Substance	Molluscicide Activity LC_10_ (48 h)	Molluscicide Activity LC_50_ (48 h)	Molluscicide Activity LC_90_ (48 h)	Molluscicide Activity LC_10_ (96 h)	Molluscicide Activity LC_50_ (96 h)	Molluscicide Activity LC_90_ (96 h)
PDAN52	92.4 ± 2.7 ppm	113.6 ± 8.4 ppm	142.0 ± 9.2 ppm	36.1 ± 7.2 ppm	79.3 ± 7.0 ppm	99.2 ± 6.5 ppm

Mortality rate of PDAN52 on the young snail *Biomphalaria glabrata* exposed for 96 h. The lethal concentration 50 (LC_50_) was 66.7 ppm and the lethal concentration 90 (LC_90_) was 114.4 ppm at 96 h. Negative control: water and 1% DMSO; positive control: niclosamide. This experiment was performed in triplicate on at least 3 different days (*n* = 9). The results expressed in the table represent the mean ± standard error.

**Table 3:** Molluscicidal effect of PDAN 52 against young *B. glabrata*.

Substance	Molluscicide Activity LC_10_ (48 h)	Molluscicide Activity LC_50_ (48 h)	Molluscicide Activity LC_90_ (48 h)	Molluscicide Activity LC_10_ (96 h)	Molluscicide Activity LC_50_ (96 h)	Molluscicide Activity LC_90_ (96 h)
PDAN52	-	-	-	-	66.7 ± 3.5 ppm	114.4 ± 3.5 ppm

Mortality rate of PDAN52 on the young snail *Biomphalaria glabrata* exposed for 96 h. The lethal concentration 50 (LC_50_) was 66.7 ppm and the lethal concentration 90 (LC_90_) was 114.4 ppm at 96 h. Negative control: water and 1% DMSO; positive control: niclosamide. This experiment was performed in triplicate on at least 3 different days (*n* = 9). The results expressed in the table represent the mean ± standard error.

**Table 4:** Molluscicide effect of PDAN 52 against *S. mansoni* cercariae.

Compound	Cercaricide Activity LC_10_ (4 h)	Cercaricide Activity LC_50_ (4 h)	Cercaricide Activity LC_90_ (4 h)
PDAN52	24.2 ± 2.8 ppm	68.0 ± 5 ppm	133.4 ± 7.3 ppm

PDAN 52 activity against *S. mansoni* cercariae for 4 h. The test was performed in triplicate on different days using a range of 80 cercariae per well during the testing of the samples. Negative control: water and 1% DMSO; positive control: niclosamide. These data are expressed as the mean ± standard error.

**Table 5:** Ecotoxicological results. The endpoints legend evaluated for each compound are defined: BCF: bioconcentration factor value; BD: biodegradation—categorizes the compounds as a positive (readily biodegradable) and as a negative otherwise; Th_pyr_pIGC50: concentration of toxicant needed to inhibit 50% growth (IGC50) of *T. pyriformis* after ca. 40 h exposure; *Daphnia*_LC50: concentration (mg/L) of compound required to kill 50% of a *D. magna* population; Minnow_LC50: concentration (mg/L) of a compound that kills 50% of a population of minnows. Andro_Filter and Estro_Filter: assess a compound’s likelihood of binding to the androgen/estrogen receptor. ADMET_Risk: identifies the potential development liabilities in drug candidates.

Compound	BCF	BD	Aquatic Toxicity			Endocrine Receptor Binding		TOX- Risk
			Th_pyr_pIGC50	*Daphnia*_LC50	Minnow_LC50	Andro_Filter	Estro_Filter	
NCL	6.65	No	1.968	1.752	3.612	Toxic	Nontoxic	2
PDAN52	25.81	No	2.426	0.057	2.564	Nontoxic	Nontoxic	2

## Data Availability

The authors declare that all data supporting the findings of this study are available within the article.
